# Clinical characteristics of coronavirus disease 2019 patients with hepatitis B virus super-infection

**DOI:** 10.1590/S1678-9946202466074

**Published:** 2024-12-16

**Authors:** Shan Yu, Cunzheng Song

**Affiliations:** 1 Shanghai Jiao Tong University School of Medicine, Ren Ji Hospital, Department of Infectious Diseases, Shanghai, China; 2 Shanghai University of Traditional Chinese Medicine, Shuguang Hospital, Shanghai, China

**Keywords:** COVID‐19, Hepatitis B virus, Liver function, Super-infection

## Abstract

COVID-19 and hepatitis B disease are significant global pandemics, both of which can lead to liver damage. This study aims to report the clinical course of liver function and disease prognosis of COVID-19 patients with hepatitis B virus (HBV) super-infections. A total of 249 outpatients with COVID-19 were enrolled in this study from December 1, 2023 to February 28, 2024. Clinical characteristics, laboratory data, chest CT findings, and patients’ treatment and outcomes were collected and analyzed retrospectively. Of the 249 outpatients, 37 (14.9%) were super-infected with HBV, whereas 212 (85.1%) showed no such outcome. This study found no significant differences between the two groups regarding age, gender, symptoms, complications, or chest CT findings. However, COVID-19 patients super-infected with HBV showed lower white blood cell, neutrophil, and platelet counts (*p* < 0.05). Additionally, total bilirubin levels were significantly higher in the SARS-CoV-2/HBV super-infected group compared to the COVID-19-only group (*p* = 0.022). After the first week of similar treatment, both groups showed almost identical outcomes, including hospitalization, severity, and mortality rates. Thus, SARS-CoV-2/HBV super-infection slightly affected liver function but did not worsen COVID-19 outcomes. Routine HBV monitoring and liver function tests are recommended to manage COVID-19 patients with HBV super-infections. This study found no clear indications of the need to change the therapeutic prescription for COVID-19 in cases of HBV super-infections.

## INTRODUCTION

The coronavirus disease 2019 (COVID-19) is an infectious disease caused by severe acute respiratory syndrome coronavirus 2 (SARS-CoV-2). About 700 million COVID-19 cases have been reported as of May 2024, including more than six million deaths in 216 countries or territories, according to the World Health Organization (WHO). The Omicron variant remains its most prevalent strain, causing recurring waves of smaller outbreaks^1^. In addition to SARS-CoV-2, the hepatitis B virus (HBV) continues to pose a public health threat worldwide. In 2019, 296 million people were estimated to live with chronic hepatitis B infection, with 1.5 million new conditions diagnosed annually, a third of which in China^2^. Patients with pre-existing liver disease constitute a high-risk population for COVID-19.

Numerous studies have determined the impact of HBV super-infection on COVID-19 and the course and prognosis between the two diseases. However, they have yielded inconsistent outcomes and primarily focused on early SARS-CoV-2 variants such as Beta and Delta, with limited research addressing Omicron-related liver injury^3–6^.

Therefore, this study aims to further explain the clinical characteristics of SARS-CoV-2 infection in patients with HBV and determine whether HBV super-infection worsens liver function deterioration and affects disease prognosis in COVID-19 patients. It ultimately aims to improve the management of this high-risk population during ongoing pandemics.

## MATERIALS AND METHODS

### Study design and participants

This retrospective, single-center study was conducted at the Infectious Diseases Department of Renji Hospital, affiliated with Shanghai Jiaotong University School of Medicine from December 1, 2022 to February 28, 2023.

Inclusion criteria: (1) a positive COVID-19 diagnosis by real-time reverse transcription-polymerase chain reaction (RT–PCR); (2) Chest CT scan and blood samples, tested and collected on the first day of consultation; and (3) treatment information and patient outcomes available for collection after one week of follow-up.

Exclusion criteria: (1) age ≤ 18 years; (2) pregnant or lactating women; and (3) patients with coinfection/super-infection with hepatitis A virus, hepatitis C virus, human immunodeficiency virus, and/or chronic liver diseases, such as primary biliary cirrhosis, liver cirrhosis, autoimmune hepatitis, and alcoholic hepatitis.

### Definitions

HBV infection was defined as a known medical history of positive for serum HBV surface antigen (HBsAg).

The severity and degree of critical condition of COVID-19 patients were assessed according to the guidelines to diagnose and treat novel coronavirus infection by the Chinese National Health Commission (Trial Version 9).

### Ethics approval and consent to participate

All subjects signed informed consent forms. This study was approved by the Research Ethics Committee of the Renji Hospital Affiliated with Shanghai Jiaotong University School of Medicine (N° LY2024-056-B).

### Data collection

Clinical characteristics, laboratory data, and radiological outcomes were collected from electronic medical records on the first visit. Treatment details and patient outcomes were obtained from electronic medical records or by telephone follow-ups on the seventh day after visit.

### Statistical analysis

Continuous variables are shown as medians (interquartile ranges [IQRs]). Categorical variables are shown as frequencies and percentages (%). Mean values and rates were compared between groups by analysis of variance or x^2^ tests. Categorical variables were compared between groups using the Mann-Whitney's U test. A *P* < .05 was considered to indicate a statistically significant difference.

## RESULTS

This study enrolled 249 patients with COVID-19, of whom 37 were super-infected with HBV. Patients’ age ranged from 18 to 80 years, with 140 (55.8%) men. The median duration from symptoms onset to clinic visit totaled one day. The most common symptoms included fever (88.4%), cough (73.9%), and fatigue (55.0%). The most common comorbidities were hypertension (23.7%), diabetes (19.3%) and cardiovascular disease (8.4%). Patients with COVID-19 and with or without HBV super-infection showed similar ages, genders, symptoms, and complications (Table 1).

**Table 1:** Clinical characteristics of patients with COVID-19/HBV super-infection.

	All (n = 249)	HBsAg− (n = 212)	HBsAg+ (n = 37)	*P* value
Age, median (IQR)	69.0 (61–74)	69.0 (61–74)	65.0 (57–73)	0.087
Male, n (%)	140 (55.8%)	118 (55.7%)	21 (56.8%)	0.901
Time from symptom onset to clinic, days	1(1–2)	1(1–2)	1(1–2)	0.464
Fever	220 (88.4%)	187 (88.2%)	33 (89.2%)	0.864
Cough	184 (73.9%)	157 (74.1%)	27 (73.0%)	0.890
Fatigue	137 (55.0%)	114 (53.8%)	23 (62.2%)	0.344
Muscle ache	47 (18.9%)	41(19.3%)	6 (16.2%)	0.654
Dyspnea	16 (6.4%)	14 (6.6%)	2 (5.4%)	0.784
Diarrhea	10 (4.0%)	8 (3.8%)	2 (5.4%)	0.641
Hypertension	59 (23.7%)	50 (23.6%)	9 (24.3%)	0.922
Diabetes	48 (19.3%)	41 (19.3%)	7 (18.9%)	0.952
Cardiovascular disease	21 (8.4%)	18 (8.5%)	3 (8.1%)	0.938
Nephrosis	20 (8.0%)	18 (8.5%)	2 (5.4%)	0.524
COPD	17 (6.8%)	16 (7.5%)	1 (2.7%)	0.281
Malignancy	14 (5.6%)	12 (5.7%)	2 (5.4%)	0.950

COPD = chronic obstructive pulmonary disease.

Among the 249 0participants, HBV-infected patients showed lower white blood cell (*P* = .034), neutrophil (*P* = 0.013), and platelet (*P* = 0.001) counts than non‐HBV patients. C-reactive protein (CRP) levels were greater than normal in COVID-19 patients with or without HBV super-infection, but the two groups of patients showed no differences (*P* = 0.406). Liver function test results showed no statistically significant differences, including alanine aminotransferase (ALT), aspartate aminotransferase (AST), gamma-glutamyl transpeptidase (GGT), or alkaline phosphatase (ALP) levels. However, SARS-CoV-2/HBV super-infected patients showed significantly higher total bilirubin (TBIL) levels than those without it (*P* = 0.022). Furthermore, the two groups showed similar chest CT findings, including bilateral lung involvement (75.9% vs. 62.2%), ground-glass opacities (36.3% vs. 32.4%), and pleural effusions (7.5% vs. 5.4%) (*P* > 0.05) (Table 2).

**Table 2:** Laboratory results for patients with COVID-19/HBV super-infection.

	HBsAg− (n = 212)	HBsAg+ (n = 37)	*P* value
CRP (mg/L)	18.9 (3.2–37.6)	16.7 (2.0–32.2)	0.406
White blood cells (×10^9^/L)	6.4 (5.0–7.8)	5.3 (4.3–7.1)	0.034*
Neutrophil (×10^9^/L)	4.2 (3.2–5.6)	3.2 (2.6–4.9)	0.013*
Lymphocyte (×10^9^/L)	1.3 (0.8–1.7)	1.5 (0.9–1.7)	0.512
Mononuclear (×10^9^/L)	0.5 (0.4–0.7)	0.6 (0.4–0.7)	0.443
Red blood cells (×10^12^/L)	4.4 (3.9–4.8)	4.5 (4.1–4.9)	0.236
Platelets (×10^9^/L)	190.5 (152.3–248.5)	152.0 (120.0–188.5)	0.001*
ALT (U/L)	25.0 (17.0–33.0)	24.0 (17.5–42.5)	0.509
AST (U/L)	36.0 (27.3–44.8)	28.0 (22.5–41.0)	0.061
GGT (U/L)	28.0 (19.0–46.8)	27.0 (16.5–60.0)	0.808
ALP (U/L)	68.5 (55.0–83.0)	73.0 (57.5–100.5)	0.155
TBIL (mmol/L)	9.8 (7.2–12.9)	11.8 (9.0–16.2)	0.022*
Chest CT Findings			
Bilateral lung involvement	161 (75.9%)	23 (62.2%)	0.078
Ground-glass opacities	77 (36.3%)	12 (32.4%)	0.649
Pleural effusions	16 (7.5%)	12(5.4%)	0.643

CRP = C-reactive protein (normal: 0-8 mg/L); ALT = alanine aminotransferase; AST = aspartate aminotransferase; GGT = gamma-glutamyl transpeptidase; ALP = alkaline phosphatase; TBIL = total bilirubin;

* P value is significant at < 0.05.

During treatment, which lasted seven days, 39 patients received oxygen therapy; 63, antiviral drugs; 143, given empirical antibiotics; and 82, corticosteroids. Treatment regimens showed no significant differences between patients with and without HBV super-infection. After one week of treatment, SARS‐CoV‐2/HBV super-infection led to hospitalization in nine (24.3%) patients, severe illness in two (5.4%) patients, and no deaths. In the COVID-19-only group, 79 (37.3%) patients were hospitalized, 16 (7.5%) developed severe illness, and five (2.4%) died. Hospitalization, severity, and mortality rates did not differ significantly between groups (Table 3).

**Table 3:** Treatments and outcomes in patients with COVID-19/HBV super-infection.

n (%)	All (n = 249)	HBsAg− (n = 212)	HBsAg+ (n = 37)	*P* value
Oxygen support	39 (15.7%)	35 (16.5%)	4 (10.8%)	0.379
Antiviral therapy	63 (25.3%)	54 (25.5%)	9 (24.3%)	0.379
Antibiotic therapy	143 (57.4%)	121 (57.1%)	22 (59.5%)	0.882
Use of corticosteroid	82 (32.9%)	74 (34.9%)	8 (21.6%)	0.113
Hospitalization	88 (35.3%)	79 (37.3%)	9 (24.3%)	0.089
Severe/critically ill	18 (7.2%)	16 (7.5%)	2 (5.4%)	0.67
Death	5 (2.0%)	5 (2.4%)	0 (0.0%)	0.345

Of the 37 COVID-19 patients who were HBsAg positive, 16 were women and 21 men. HBV DNA occurred in 29 patients, with 11 having levels above 20 IU/mL. Of the 37 patients, two had cirrhosis, and 14 received nucleoside analogs (oral entecavir, 0.5 mg once daily) during this retrospective investigation (Table 4).

**Table 4:** Hepatitis B serological marker and nucleoside analogue use in COVID-19 patients super-infected with HBV.

Patient	Age (years)	Sex	HBsAg	Anti-HBs	HBeAg	Anti-HBe	Anti-HBc	HBV-DNA	Use of nucleoside analogue
			(Pos/Neg)	(Pos/Neg)	(Pos/Neg)	(Pos/Neg)	(Pos/Neg)	(IU/mL)	
1	35	male	Pos	Neg	Pos	Neg	Pos	**3.17E+01**	
2	58	male	Pos	Neg	Neg	Pos	Pos	<20	Yes
3	76	female	Pos	Neg	Pos	Neg	Pos	**3.40E+01**	
4	56	female	Pos	Neg	Neg	Pos	Pos	**2.75E+02**	
5	46	male	Pos	Neg	Pos	Neg	Pos	<20	
6	69	female	Pos	Neg	Pos	Neg	Pos	**2.22E+02**	Yes
7	76	female	Pos	Neg	Pos	Neg	Pos	**1.63E+02**	Yes
8	66	female	Pos	Neg	Neg	Pos	Pos	<20	Yes
9	71	female	Pos	Neg	Pos	Neg	Pos	**3.34E+03**	
10	74	male	Pos	Neg	Neg	Pos	Pos	<20	Yes
11	73	male	Pos	Neg	Neg	Pos	Pos	**4.33E+01**	
12	71	male	Pos	Neg	Neg	Pos	Pos	**2.51E+03**	
13	75	female	Pos	Neg	Neg	Pos	Pos	NA	
14	63	female	Pos	Neg	Neg	Pos	Pos	NA	
15	74	male	Pos	Neg	Neg	Pos	Pos	<20	
16	75	male	Pos	Neg	Neg	Pos	Pos	**5.52E+02**	Yes
17	64	male	Pos	Neg	Neg	Pos	Pos	**2.03E+03**	Yes
18	71	male	Pos	Neg	Neg	Pos	Pos	<20	
19	41	male	Pos	NA	NA	NA	NA	NA	
20	74	female	Pos	Neg	Neg	Pos	Pos	<20	Yes
21	38	male	Pos	NA	NA	NA	NA	NA	
22	65	female	Pos	Neg	Neg	Pos	Pos	**1.71E+01**	
23	61	male	Pos	Neg	Neg	Pos	Pos	NA	
24	59	female	Pos	Pos	Neg	Pos	Pos	NA	
25	74	female	Pos	Neg	Neg	Pos	Pos	<20	
26	46	female	Pos	Neg	Neg	Pos	Pos	<20	Yes
27	71	male	Pos	Neg	Neg	Pos	Pos	**1.31E+06**	Yes
28	78	male	Pos	Neg	Neg	Pos	Pos	**4.02E+03**	
29	65	female	Pos	Neg	Neg	Pos	Pos	NA	
30	62	male	Pos	Neg	Neg	Pos	Pos	<20	
31	68	male	Pos	Neg	Neg	Pos	Pos	**3.72E+02**	
32	55	male	Pos	Neg	Neg	Pos	Pos	NA	
33	64	male	Pos	Neg	Neg	Pos	Pos	**9.23E+02**	
34	63	male	Pos	Neg	Neg	Pos	Pos	<20	Yes
35	46	female	Pos	Neg	Neg	Pos	Pos	**2.16E+02**	Yes
36	57	male	Pos	Pos	Neg	Pos	Pos	**5.15E+02**	Yes
37	49	female	Pos	Neg	Neg	Pos	Neg	**9.29E+01**	Yes

The bold text indicates a value of >20 IU/mL HBV-DNA, which was considered positive; NA = data not available; Pos = positive; Neg = negative.

## DISCUSSION

SARS-CoV-2 and HBV significantly threaten public health and contribute to the global burden of infectious diseases. Both viruses cause varying degrees of liver damage^7,8^. This study aimed to better understand the risks associated with SARS-CoV-2/HBV super-infection and to improve the management of these patients during ongoing epidemics.

This study systematically compared the clinical characteristics, hematological parameters, serum biochemical indices, and chest CT findings of COVID-19 patients with and without HBV super-infection, observing that white blood cell, neutrophil, and platelet counts were lower in COVID-19 patients super-infected with HBV than in those without HBV infection. The Hunan Provincial Centre for Disease Control and Prevention reported similar findings, finding generally normal or reduced total white blood cell, neutrophil, and platelet counts^9^. Previous studies have shown that viral suppression in hepatitis B patients led to hematopoietic dysfunction and immunoinflammatory responses, resulting in abnormal blood counts^10,11^. Thus, super-infection with SARS-CoV-2 and HBV might exacerbate hematological abnormalities. Moreover, this study found that elevated CRP levels in COVID-19 patients, regardless of HBV super-infection status, with no significant differences between groups. This suggested that while the bodily response to SARS-CoV-2 infection was robust, HBV super-infection might have little impact on CRP levels in COVID-19 patients.

In our study, the levels of ALT and AST, which were directly related to liver function, did not significantly differ between groups. However, TBIL levels, which were closely related to bilirubin metabolism and cholestasis, were significantly higher in SARS-CoV-2/HBV super-infected patients. This was identical to the findings in Chen *et al.* ^12^. Angiotensin converting enzyme 2, a major receptor mediating the entry of SARS-CoV-2 into human cells, is expressed in type II alveolar epithelial cells and in cholangiocytes (in high levels)^13,14^. Recent studies analyzing online single-cell RNA sequencing data from healthy liver tissues also confirmed that the level of angiotensin converting enzyme 2 mRNA in bile duct cells was comparable to that in ATII cells but not in hepatocytes^15,16^. These findings suggest that SARS-CoV-2 could infect bile duct cells, leading to liver function abnormalities in patients. Moreover, previous research has indicated that elevated TBIL levels in HBV patients might result from impaired hepatic uptake, binding, and elimination of bilirubin due to HBV^17^. We assumed that the level of TBIL would be abnormal under the dual influence of HBV and SARS-CoV-2. However, we found no direct and forceful evidence that the presence of HBV infection exacerbated liver damage in COVID-19 patients.

Previous studies showed that COVID-19 pneumonia tended to manifest on lung CT scans as bilateral ground-glass opacities and that the presence of pleural effusion was a poor prognostic indicator in patients infected with MERS-CoV^18,19^. This study observed no significant differences in the CT features of COVID-19 between patients with HBV super-infection and those without it, suggesting that super-infection with SARS-CoV-2 and HBV would not significantly impact lung infections.

Finally, this study also compared the first week of treatments and outcomes of COVID-19 patients with and without HBV infection. Under almost identical treatments, SARS-CoV-2/HBV super-infection did not affect the course or prognosis of COVID-19, including the rates of hospitalization severe illness and mortality.

## CONCLUSION

In summary, this study provided insights into the clinical characteristics and chest CT findings of patients super-infected with SARS-CoV-2 and HBV. It showed that HBV super-infection slightly affected liver function, but it did not impact liver function and the overall outcome of COVID-19. Routine HBV monitoring and liver function tests, instead of changing or adding the therapeutic prescription, are recommended for managing COVID-19 patients with HBV super-infection.
